# Parenting in a 24/7 Economy: Mothers’ Non-standard Work Schedules and Involvement in Children’s Education

**DOI:** 10.3389/fpsyg.2022.905226

**Published:** 2022-07-07

**Authors:** Minseop Kim, Nahri Jung, Larasati Wulandari

**Affiliations:** ^1^Department of Social Work, The Chinese University of Hong Kong, Shatin, Hong Kong SAR, China; ^2^Department of Economics, University of Toronto, Toronto, ON, Canada

**Keywords:** maternal employment, non-standard work schedules, non-standard hours, weekend work, parental involvement, parental engagement, shift work

## Abstract

Evidence suggests that parental involvement in children’s education has a positive impact on children’s educational achievements and wellbeing outcomes. The trend toward 24/7 economies has obliged many mothers to work non-standard schedules (i.e., schedules outside traditional daytime, Monday–Friday work schedules). This has raised concerns over how non-standard work schedules affect parenting behaviors, including paternal involvement in children’s education. Using data from mothers of young children (age 5–6) in Hong Kong (*N* = 433), this study examined the association between maternal work schedules and involvement in children’s home and school activities. The results of structural equation modeling found that weekend work was associated with lower levels of home-based involvement. By contrast, working non-standard hours was linked with higher levels of school-based involvement. This pattern suggested that non-standard work schedules could make it either easier or harder for mothers to balance work and family responsibilities, depending on the circumstances (i.e., whether they work non-standard hours or weekends). These findings, indicating that the effects of non-standard work schedules are not uniformly negative or positive, warrant sophisticated policy efforts to address the potential adverse effects of non-standard work schedules and avoid undermining their potential to be used as a family-friendly option.

## Introduction

In an attempt to improve the academic achievements of children, the importance of parental involvement in children’s education has attracted a considerable attention from policy makers, educators, and parents (Slicker et al., 2021; Jeynes, 2022). Parental involvement, defined broadly as parents’ investment in the education of their children (LaRocque et al., 2011), typically involves parental behaviors in home and school settings, suggesting its two subtypes (Boonk et al., 2018): (1) home-based involvement (i.e., activities carried out at home for children’s learning, such as assisting with children’s homework), and (2) school-based involvement (i.e., activities that require parents to have contact with schools, such as attending school events or parent–teacher conferences). Accumulating evidence suggests that both forms of parental involvement, despite their distinctive nature, produce positive effects on children’s academic achievement across grade levels (Wilder, 2013; Boonk et al., 2018; Kim, 2019b), while also helping to prevent truancy and school dropout (Paul et al., 2021). Their positive outcomes have also been observed for other child wellbeing indicators, such as health and mental health (Wang and Sheikh-Khalil, 2014), and socio-emotional competence (Sheridan et al., 2019). As a result, parental involvement initiatives have been a key component of social policies in developed countries. A typical example is the No Child Left Behind Act in the United States, which identified parent involvement as one of six reform areas to enhance children’s academic achievement and overall wellbeing (Epstein, 2010; Berkowitz et al., 2021).

### Maternal Employment and Involvement in Children’s Education

The rate of parental involvement is often discussed in relation to the working condition of parents (Hook and Wolfe, 2013; Genadek and Hill, 2017), particularly mothers (Haley-Lock and Posey-Maddox, 2016). Although there has been some erosion of the gender division of labor (Buchanan et al., 2020), mothers often continue to assume the main responsibility for teaching their children (Chao, 2022). Notably, maternal working hours have been identified as a factor in creating a barrier to parental involvement (Youn et al., 2012), given that mothers who are working long hours are likely to experience time poverty (i.e., the lack of adequate time to work and be involved in children’s education) (Chin and Newman, 2002). While the impact of working hours on family life has long been of concern (Ganster et al., 2016; Prickett and Augustine, 2021), a mere handful of empirical studies have validated the link between mother’s working hours and their involvement in children’s education. In an early study of mothers with adolescents in the United States, for instance, Muller (1995) found that mothers employed full time were less likely to check homework and volunteer at school than part-time working mothers or those who were not in work. Interestingly, part-time working mothers were found to have higher levels of involvement than those not in work, suggesting that the association between maternal working hours and parental involvement might be non-linear. Later studies, examining mothers with younger children (e.g., kindergarteners), documented similar findings, as Weiss et al. (2003) reported that mothers employed part-time had the highest level of school-based involvement. Youn et al. (2012) also documented part-time working mothers to be the most involved, while such a non-linear association was more salient with school-based involvement (than with home-based involvement), suggesting that the effect of maternal working hours may differ by the types of maternal involvement in children’s education.

### Non-standard Work Schedules

While these studies were important, they commonly assumed that mothers work a fixed, regular daytime schedule, which is no longer necessarily true. As a 24/7 economy has evolved the temporal pattern of working hours has become increasingly diverse (Presser, 2003; Bolino et al., 2021), which may have distinct implications for maternal involvement in children’s education (Vincent and Neis, 2011). Nowadays non-standard work schedules, encompassing both working non-standard hours (e.g., evening or night shifts) and non-standard days (e.g., weekends), have become standard. In the United States, for instance, about one-fifth of all employees work in the evening, at night, on a rotating shift, or at irregular hours (McMenamin, 2007), while 30% of employees work on Saturdays, Sundays, or public holidays (Bureau of Labor Statistics, 2019). Similar point-in-time estimates were reported in Canada (Williams, 2008) and Australia (Dockery et al., 2009), while a study, adopting life-time perspectives (Presser and Ward, 2011), found that nearly 90% of American employees experienced a non-standard schedule over the course of their worklife. Working outside of weekday, daytime hours is also pervasive among parents with dependent children. A study by Davis et al. (2006) revealed that over 50% of dual earner families with adolescent children had at least one parent working evenings, nights, or on weekends. This striking finding underscores the importance of determining whether non-standard work schedules affect maternal involvement in children’s education, and if so, how.

Work-family conflict theory, which highlights the inevitable inter-role conflicts faced by working parents (Greenhaus and Beutell, 1985), suggests that non-standard work schedules may interfere with parental roles through time-based and strain-based conflicts (Moilanen et al., 2019; Taiji and Mills, 2019). Time-based conflict may arise when non-standard work schedules lead to structural desynchronization in work time and family time, suggesting that non-standard schedules may reduce the time overlap that mothers can share with their children (Han et al., 2010; Enchautegui, 2013). For instance, working evening or night hours may make it difficult for mothers to supervise their children’s development-related activities at home (e.g., helping homework assignment, and supervise screen-based time), due to misalignment between their work schedules and children’s time after school hours (Tsai, 2022). Similarly, working rotating shifts or irregular hours may disrupt mothers’ ability to maintain home-based and school-based involvement, due to the variable or unpredictable nature of the schedules (Kim and Liu, 2021). Weekend work could be disruptive as well in that parents often use weekends for parental involvement activities that require significant time investment, or for leisure and sport activities (Craig and Brown, 2014). In addition to time-based conflict, strain-based conflict stemming from non-standard work schedules may limit the level of physical and emotional availability for parental involvement (Vincent and Neis, 2011). It has been demonstrated that non-standard work schedules place workers at a higher risk of sleeping problems, stress, chronic fatigue, and depression (Sato et al., 2020; Pereira et al., 2021), due to the disruption of the physiologic rhythms (Vogel et al., 2012). Such adverse health effects suggest that non-standard schedules may undermine mothers’ capacity to be responsive and sensitive to their children’s needs (Grzywacz et al., 2011), which in turn may reduce maternal engagement with children in educational activities (Gassman-Pines, 2011). In other words, non-standard work schedule may reduce maternal physical and mental capacity for maternal involvement, even when parents are able to find the time for maternal involvement activities (Vincent and Neis, 2011).

Although previous studies have often highlighted the negative aspects of non-standard work schedules (Bolino et al., 2021), these schedules can also have a potentially positive impact on maternal involvement. According to work-family enrichment theory (Greenhaus and Powell, 2006), maternal employment, even if it involves atypical schedules, may offer resources that help mothers achieve their parental roles. For example, material resources (e.g., money) obtained from maternal employment can be used to enhance the rate and quality of parents’ educational involvement through the purchase of goods and services (Hill et al., 2016). More importantly, non-standard work schedules are sometimes seen as family-friendly work options that enable mothers to devote more time to family responsibilities (Kim, 2020b). For instance, evening or night work may enable mothers, particularly those with young children, to stay at home during daytime. This may allow them to spend more time with their children, possibly enhancing their educational involvement at home and school. In this regard, a recent study by Kim (2020b) revealed that approximately one third of American shift workers indeed chose to work non-standard schedules on a voluntary basis. In particular, mothers of young children were more likely to make such choices, suggesting that they may attempt to manage their role commitments by working non-standard schedules.

### Current Study

These disparate perspectives underscore the importance of *empirical* investigations to determine whether and how maternal non-standard work schedules influence maternal involvement in children’s education. An examination of work schedules and working hours will also provide a fuller picture of the association between maternal employment and educational involvement. In Chinese contexts, however, efforts to unpack the interplay between work characteristics and parenting behaviors have been scant, despite the implications of this interplay for Chinese parents who juggle work and family responsibilities. For example, Hong Kong, like other developed countries, has seen a gradual increase in the participation of women in the labor force, from 47.9% in 1991 to 54.2% in 2020 (Census and Statistics Department, 2021). While this increase has largely been driven by an influx of mothers into the labor market (Lau et al., 2006; Tong and Chiu, 2016), the economic reality of Hong Kong (e.g., the high cost of living) often requires them to make a strong commitment to work roles (e.g., working long hours), which leads them to commonly experience high levels of work-family conflict (Jung and Kim, 2021). The expansion of Hong Kong’s service economy has also speeded the development of a 24/7 economy (Presser, 2003), in which a substantial proportion of employees work non-standard schedules. In a recent study of dual earner families with young children in Hong Kong, Kim (2021) reported that 14.4% of mothers worked non-standard hours. Such employment trends, paired with a body of evidence showing the positive impacts that parental involvement can produce in Chinese contexts (Lau et al., 2011; Wu and Sun, 2020), warrant systematic investigation to determine the association between mothers’ work characteristics and their involvement in children’s education. The present study therefore sought to examine whether various maternal work characteristics, including the number of working hours, working non-standard hours, and working on weekends, affect maternal involvement among mother of young children in Hong Kong, and if so, how.

## Materials and Methods

### Data and Sample

Approximately 90% of children aged 5–6 attend kindergartens in Hong Kong. In order to collect representative data of mothers with young children, this study used the Hong Kong Government Education Bureau’s district kindergarten list as a sample frame. It listed all registered kindergartens in Hong Kong. Firstly, the kindergartens were randomly selected in such a way to ensure socioeconomic representativeness. Hong Kong’s 18 districts were divided into 3 areas based on the median household income of the districts: low-income districts, middle-income districts, and high-income districts. Accounting for the number of preschools in each area, 13, 10, and 15 kindergartens were randomly chosen from the low-, middle-, and high-income areas, respectively. Secondly, parents of the final-year preschoolers (5- to 6-year-old) in the selected kindergartens were invited to participate in the study, following approval from the kindergartens’ principals. Among 615 families who expressed interest and thus received the survey questionnaires, 521 returned the survey questionnaire (response rate = 84.7%), *via* postal systems in January–February, 2019. While the primary caregiver of the child was asked to complete the survey, 433 respondents were identified as mothers with valid responses on parental involvement in children’s education, and selected for data analyses. All the participating families received a supermarket coupon (100 HK$ ≈ 12 US$) as a reward for their participation.

### Measures

#### Maternal Involvement

Maternal involvement in children’s education was measured by the Early Parental Involvement Scale (EPIS) (Lau et al., 2011). The 26-item scale is designed to assess 6 dimensions of parental involvement, based on parents’ self-report on their involvement behaviors using a 5-point Likert scale. Of the six sub-scales of EPIS, four subscales reflect parents’ home-based involvement: (1) Parent Instruction (parents’ direct instruction to promote children’s self-care ability and socio-emotional development, seven items); (2) Homework Involvement (parents’ engagement in monitoring and assisting their children’s completion of school work, three items); (3) Parent Discussion (parents’ conversation with their children about issues related to primary school, such as teachers and routines in primary school, five items); (4) Home Learning Activities [parents’ participation in home learning activities (e.g., reading stories) for language and cognitive development, five items]. The other two subscales, assessing school-based involvement include (1) Home–School Conferencing (parents’ communication with the school, four items), and (2) School Involvement (parents’ participation in school activities as volunteers and/or administrators, two items). EPIS has demonstrated its reliability and validity in samples of parents with preschoolers in Hong Kong and mainland China (Lau et al., 2011).

#### Hourly Work Schedules

Based on reviews by Lambert and Henly (2014), who compared diverse approaches for measuring non-standard work schedules, mothers were asked to indicate the schedule they usually worked at their main job over the past 3 months, using the following categories: (1) standard (if the job primarily begins at 8 a.m. or later and ends by 6 p.m.); (2) evenings (if a mother works primarily between 6 p.m. and midnight); (3) nights (if a mother works primarily between midnight and 8 a.m.); (4) rotating shift (if the work shift changes periodically from days to evenings/nights or vice versa); (5) irregular or varying hours; and (6) other schedules, and (7) not working. However, preliminary analyses found that the detailed categories caused small cell/frequency issues in some categories. We therefore followed the practice used in a previous study (Odom et al., 2013) of merging the categories of evening, night, rotating shifts, irregular hours, and others into a single category. Our work schedule variable therefore consisted of three categories: (1) standard hours, (2) non-standard hours, and (3) not working.

#### Weekend Work

Mothers were asked how often they worked on weekends during the past 3 months. The ordinal variable consists of the following categories: “never (i.e., working weekdays only),” “rarely,” “sometimes,” “often,” “always,” and “not working.”

#### Working Hours

Mothers’ working hours were assessed by a categorical variable reflecting usual weekly working hours (at all jobs) over the past 3 months. The categorical variable distinguished “not working (0 h),” “part-time work (1–29 h),” “full-time work (30–47 h),” “long working hours (48–59 h),” “very long working hours (60 h or more).” These distinctions reflect the working hour classification systems used in the Hong Kong Census and public/government reports (Census and Statistics Department, 2010; Legislative Council Secretariat, 2019).

#### Control Variables

This study controlled for an extensive set of maternal, child, and family characteristics that may be correlated with both levels of parental involvement (Sy et al., 2007), and maternal work characteristics (Presser, 1995; Kim, 2020b). The maternal control variables included highest education levels (<high school, high school, and >high school), and age (as of the survey year), while the family characteristics included family income (monthly), total number of children under 18, family structure (two-parent vs. single-parent family), presence of a domestic helper at home (Yes vs. No), and presence of one or more grandparents at home (Yes vs. No). The child characteristics included the child’s gender (male vs. female), age (5-year-old vs. 6-year-old), and ethnicity (Chinese vs. non-Chinese), and general health assessed by the mother’s response to a single question (“In general, would you say the child’s health is —?”) on a 5-point Likert scale (1: poor – 5: excellent). In data analysis, child health was treated as a continuous variable, instead of a categorical variable, to reduce model complexity.

### Data Analysis

**FIGURE 1:**
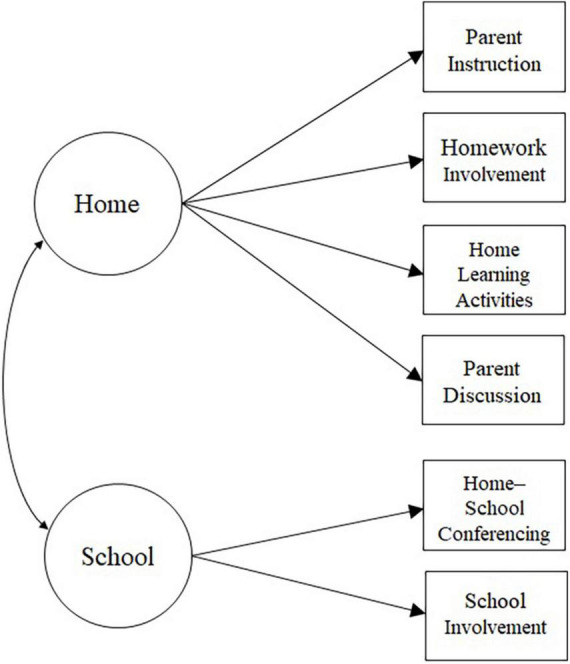
Measurement model.

**FIGURE 2:**
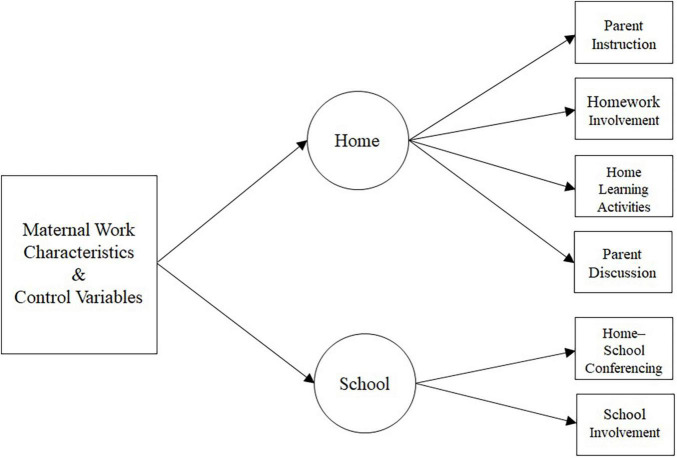
Structural model.

Structural equation modeling (SEM) was used to examine the association between mothers’ work characteristics and their home-based and school-based involvement in children’s education. SEM, unlike conventional linear regression, is able to deal with measurement errors by treating parental involvement as a latent variable. It also allows us to examine two types of parental involvement (i.e., home-based involvement and school-based involvement) simultaneously. Firstly, confirmative factor analysis (CFA) was conducted to test whether the measurement model of parental involvement fits the data well. In an effort to reduce model complexities (and thereby enhance the stability of parameter estimates), CFA relied on an item-parceling approach that uses a sum of multiple items that measure the same sub-construct as an indicator for higher-level constructs. In other words, total scores for four subscales pertaining to home-based involvement were used as indicators for a latent variable of home-based involvement, while two subscale scores were used to measure school-based involvement (Figure 1). To assess model fit, model Chi-square was examined. Given the fact that model Chi-square is sensitive to large samples, additional fit indices, including the comparative fit index (CFI), the root mean square error of approximation (RMSEA), and the standardized root mean square residual (SRMR), were also assessed. A CFI value of >0.95 indicates a close fit, while values in the 0.90–0.95 range are considered acceptable (Hu and Bentler, 1999). For RMSEA and SRMR, a value of <0.05 indicates a close fit, while <0.08 is considered reasonable (Browne and Cudeck, 1993; Hu and Bentler, 1999). The measurement model tested by CFA was subsequently used to estimate a structural model in which maternal work characteristics predict the latent variables of home-based and school-based involvement, controlling for maternal, family, and child characteristics (Figure 2). All models were estimated by maximum likelihood with robust standard errors (i.e., MLR) to deal with potential non-independence issues that may result from the use of multi-stage sampling methods (i.e., mothers/children nested within kindergartens). Regarding missing data, each variable had a few missing data (in total, less than 2% of the cases). To deal with this missing data, full information maximum likelihood (FIML) methods were used. All of the data analyses were conducted, using the *lavaan* package (version 0.6-10) in R (version 4.1.2).

## Results

### Descriptive Statistics

Table 1 presents the descriptive statistics of the sample. More than half of the mothers (55.76%) were employed. Of those employed, 14.35% worked in a job with non-standard hours. Working varying/irregular hours was the most common type (55.86% of mothers working non-standard hours, or equivalently 8.02% of working mothers), followed by evening work, rotating shift, other shifts, and night work. Weekend work was even more pervasive. Of working mothers, only 27.43% had regular Monday–Friday schedules, indicating that about three fourths of working mothers had at least some experience of weekend work. Significantly, 7.59% of working mothers always worked during the weekends. Working long hours was also common: 43.46% of working mothers worked 48 h or more weekly. The average age of the mothers was 37.67 (SD = 4.96), and approximately 40% had at least some college education. They were predominantly living with a spouse or domestic partner (90.72%). Average monthly family income was approximately HK$48,900. About one third had domestic helpers (32.48%), or lived with grandparent(s) at home (29.60%). The children were predominantly Chinese (96.5%). 54.04% of the children were boys.

**TABLE 1:** Descriptive statistics.

	% (% in working mothers)	Mean (SD)
Mother’s employment	55.76%	
**Hourly work schedule (ref: standard)**	47.76% (85.65%)	
Non-standard	8.00% (14.35%)	
*Evening*	1.41% (2.53%)	
*Night*	0.47% (0.84%)	
*Rotating*	0.94% (1.69%)	
*Varying/irregular*	4.47% (8.02%)	
*Others*	0.71% (1.27%)	
Not working	44.24%	
**Weekend work (ref: never)**	15.29% (27.43%)	
Rarely	13.88% (24.89%)	
Sometimes	14.35% (25.74%)	
Often	8.00% (14.35%)	
Always	4.24% (7.59%)	
Not working	44.24%	
**Working hours [ref: part-time (0–29)]**	8.47% (15.19%)	
Full-time (30–47)	23.06% (41.35%)	
Long (48–59)	16.71% (29.96%)	
Very long (60 or more)	7.53% (13.50%)	
Not working (0)	44.24%	
**Mother education (ref: <high school)**	21.06%	
High school diploma	37.04%	
>High school	41.90%	
**Family structure (ref: two-parent)**	90.72%	
Single-parent	9.28%	
**Child age (ref: 6 years old)**	12.47%	
5 years	87.52%	
**Child gender (ref: female)**	45.96%	
Male	54.04%	
**Child ethnicity (ref: non-Chinese)**	3.50%	
Chinese	96.50%	
**Living with grandparent(s)**	29.60%	
**Living with domestic helper(s)**	32.48%	
**Mother age**		37.67 (4.96)
**Number of children**		1.84 (0.67)
**Family income (in HK$10,000)**		4.89 (6.83)
**Child health**		3.27 (0.79)

### Confirmative Factor Analysis Results

**FIGURE 3:**
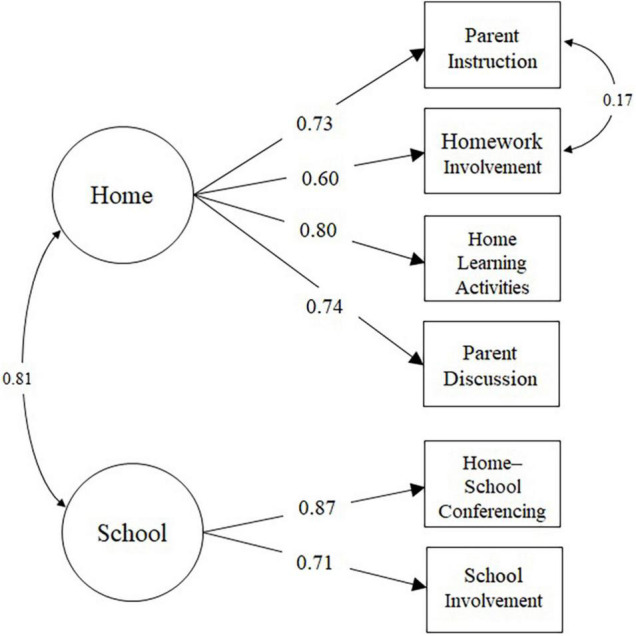
Confirmative factor analysis model.

Confirmative factor analysis found that most fit indices for the initial two-factor model (Figure 3) met the criteria for adequate measurement models [χ^2^ = 30.92(8), *p* < 0.001; CFI = 0.974; SRMR = 0.032], except the RMSEA (=0.082; 90% CI = 0.055–0.110), suggesting the initial model had a slight misfit to the data. Modification indices showed that model fit would improve if the residuals for two indicators (“Parent Instruction” and “Homework Involvement”) are correlated. Given that the two indicators are logically linked and designed to assess the same latent construct, the initial model was revised to add the residual correlation (Figure 3). The revised model fitted the data adequately [χ^2^ = 24.74(7), *p* < 0.001; CFI = 0.980; SRMR = 0.027; RMSEA = 0.077, 90% CI = 0.048–0.108], with factor loadings that were statistically significant (*p* < 0.001) in the expected direction. Satorra–Bentler scaled Chi-square difference tests (Satorra and Bentler, 2010) also revealed that the model fit of the revised model was significantly better (*p* < 0.05) than that for the initial model. As a result, the revise model was used for the structural model.

### Structural Equation Modeling Results

For ease of model comparison, SEM results are presented in Table 2, instead of in the form of path diagrams. Model 1, examining a binary variable of maternal employment (employed vs. not employed), found that employed mothers had lower levels of home-based (*b* = −1.597, *p* < 0.001) and school-based involvement (*b* = −1.551, *p* < 0.001) than mothers not in the labor force. To see if maternal working conditions further differentiate maternal involvement among working mothers, Model 2 explored the categorical variable of weekly working hours as a predictor. Long working hours were generally associated with lower levels of maternal involvement, with mothers working long hours (48–59 h a week) having significantly lower levels of home-based involvement than part-timers (*b* = −1.327, *p* < 0.05). Model 3, investigating the hourly timeframe of work schedule, found that there was no difference in home-based involvement between mothers working standard daytime hours and those working non-standard hours. For school-based involvement, however, mothers working non-standard hours had greater levels than those working standard hours (*b* = 1.177), though the difference was marginally significant (*p* < 0.1). In results not shown (but available on request), Model 3 was estimated again, with the reference category changed to those not in the labor force. In the model, there was no significant difference between mothers working non-standard hours and those not in work (*b* = −0.626, *p* > 0.05), indicating that the level of school-based involvement among mothers working non-standard hours was comparable to that for full-time mothers (i.e., mothers not in work). Model 4 examined weekend work as a predictor. The model found that by and large weekend workers had lower levels of home- and school-based involvement than working mothers who worked Monday–Friday only (i.e., working mothers who *never* worked on weekends). In particular, mothers who *often* worked on weekends had lower levels of home-based involvement (*b* = −1.784, *p* < 0.05) than working mothers who worked Monday–Friday only. This negative association was absent in the case of those who *always* worked weekends. Finally, Model 5 examined working hours, hourly work schedules, and weekend work simultaneously. It is worth noting that the model was estimated among working mothers (i.e., the sample excluding mothers not in the labor force), because the three predictors of maternal work characteristics had a common category, namely “not working,” which caused convergence failures when the model was estimated for the total sample of mothers. Consistent with Model 3 and Model 4, Model 5 confirmed that mothers who often worked on weekends had significantly lower levels of home-based involvement than working mothers who worked weekdays only, while mothers working non-standard hours had higher levels of school-based involvement than those working standard hours. In contrast, working hours was found to be a non-significant predictor of maternal involvement, indicating that the association between working long hours and home-based involvement (detected in Model 2) lost its significance after adjustment for work schedule characteristics.

**TABLE 2:** SEM results.

	Model 1				Model 2				Model 3			
	Home		School		Home		School		Home		School	
	*b*	SE	*b*	SE	*b*	SE	*b*	SE	*b*	SE	*b*	SE
**Employment**												
Employed	−1.597	0.353***	−1.551	0.345***								
Not working	Ref		Ref									
**Working hour**												
Part-time (1–29)					Ref		Ref					
Full-time (30–47)					−0.535	0.601	−0.312	0.689				
Long (48–59)					−1.327	0.650*	−0.950	0.712				
Very long (60 or over)					−1.019	0.779	−0.968	0.778				
Not working (0)					0.919	0.600	1.070	0.637^†^				
**Hourly work schedules**												
Standard									Ref		Ref	
Non-standard									−0.075	0.689	1.177	0.636^†^
Not working									1.592	0.358***	1.803	0.346***
**Weekend work**												
Never												
Rarely												
Sometimes												
Often												
Always												
Not working												
**Mother’s age**	0.077	0.028**	0.043	0.029	0.081	0.029**	0.047	0.029	0.077	0.028**	0.043	0.029
**Mother’s education (ref: <HS)**												
High school diploma	−0.494	0.412	−0.043	0.389	−0.449	0.411	−0.017	0.386	−0.498	0.409	−0.045	0.385
>High school	−0.761	0.429^†^	−0.446	0.434	−0.719	0.427^†^	−0.426	0.434	−0.756	0.428^†^	−0.428	0.425
**Child gender (ref: female)**	−0.039	0.310	−0.058	0.295	−0.013	0.306	−0.042	0.295	−0.038	0.309	−0.035	0.290
**Child age (ref: 6 years old)**	−0.375	0.488	−0.159	0.425	−0.384	0.484	−0.168	0.425	−0.378	0.487	−0.125	0.421
**Child ethnicity (ref: non-Chinese)**	−0.308	0.616	−0.151	0.575	−0.063	0.587	−0.002	0.579	−0.306	0.612	−0.247	0.585
**Child health**	0.758	0.200***	0.660	0.195**	0.731	0.202***	0.642	0.194**	0.759	0.199***	0.650	0.192**
**Family income (in 10,000 HK$)**	0.038	0.016*	0.013	0.019	0.042	0.016*	0.016	0.019	0.038	0.016*	0.015	0.019
**Number of children**	−0.578	0.263*	−0.295	0.235	−0.631	0.262*	−0.340	0.232	−0.578	0.263*	−0.337	0.230
**Single-parent family**	−0.392	0.558	0.084	0.541	−0.393	0.565	0.078	0.551	−0.383	0.556	0.107	0.530
**Grandparent(s)**	−0.141	0.349	0.077	0.340	−0.145	0.349	0.078	0.338	−0.152	0.345	0.119	0.335
**Domestic helper(s)**	0.226	0.358	−0.289	0.385	0.364	0.348	−0.187	0.389	0.226	0.360	−0.168	0.374
*R* ^2^	0.140		0.140			0.151		0.150	0.141		0.156	
Model Chi-square	147.501 (59)***					160.895 (71)***					163.664 (63)***	
CFI	0.921					0.918					0.911	
RMSEA (90% CI)	0.059 (0.047–0.071)					0.054 (0.043–0.065)					0.061 (0.049–0.072)	
SRMR	0.030					0.028					0.030	
AIC	22977.19					23348.906					23054.609	
BIC	23111.784					23531.954					23204.457	
*N*	433					433					433	

	**Model 4**				**Model 5**			
	***Home***		***School***		***Home***		***School***	
	**b**	**SE**	**b**	**SE**	**b**	**SE**	**b**	**SE**

**Employment**								
Employed								
Not working								
**Working Hour**								
Part-time (1-29)					Ref		Ref	
Full-time (30-47)					−0.148	0.569	−0.218	0.641
Long (48-59)					−0.761	0.611	−0.790	0.653
Very Long (60 or over)					−0.469	0.693	−0.751	0.705
Not working (0)								
**Hourly Work Schedules**								
Standard					Ref		Ref	
Nonstandard					−0.308	0.573	1.315	0.561*
Not working								
**Weekend Work**								
Never	Ref		Ref		Ref		Ref	
Rarely	−0.873	0.515†	0.056	0.523	−0.688	0.469	0.309	0.483
Sometimes	−0.754	0.542	−0.472	0.528	−0.231	0.478	−0.237	0.472
Often	−1.784	0.629**	−0.580	0.587	−1.242	0.566*	−0.886	0.555
Always	0.462	0.878	0.411	1.051	0.645	0.681	0.236	0.857
Not working	0.986	0.448*	1.398	0.456**				
**Mother’s Age**	0.073	0.029*	0.043	0.029	0.034	0.036	0.016	0.040
**Mother’s Education (ref: < HS)**								
High School Diploma	−0.445	0.406	−0.037	0.389	0.300	0.570	0.500	0.652
> High School	−0.668	0.429	−0.396	0.436	−0.121	0.558	0.220	0.642
**Child Gender (ref: Female)**	−0.087	0.311	−0.090	0.300	−0.070	0.327	−0.172	0.348
**Child Age (ref: 6 years old)**	−0.457	0.498	−0.166	0.427	−0.218	0.503	−0.166	0.521
**Child Ethnicity (ref: Non-Chinese)**	−0.109	0.591	−0.103	0.592	−0.214	0.518	−0.917	0.828
**Child Health**	0.734	0.199***	0.641	0.196**	0.707	0.209**	0.231	0.209
**Family Income (in 10,000 HK$)**	0.034	0.015*	0.010	0.019	0.034	0.017*	0.014	0.018
**Number of Children**	−0.621	0.261*	−0.310	0.237	−0.444	0.266†	−0.221	0.289
**Single-parent family**	−0.386	0.536	0.082	0.534	−0.150	0.662	0.164	0.845
**Grandparent(s)**	−0.137	0.347	0.118	0.338	−0.011	0.335	0.000	0.385
**Domestic Helper(s)**	0.325	0.358	−0.267	0.379	0.509	0.323	−0.312	0.393
*R* ^2^	0.162		0.145		0.162		0.104	
Model Chi-Square	158.504 (75)***				139.118(87)***			
CFI	0.925				0.911			
RMSEA (90% CI)	0.051 (0.040-0.062)				0.046(0.031-0.060)			
SRMR	0.027				0.024			
AIC	23249.075				16650.865			
BIC	23450.069				16791.488			
N	433				286			

^†^p < 0.1, *p < 0.05, **p < 0.01, ***p < 0.001.

## Discussion

In an era when many mothers are employed, understanding how different aspects of maternal work influence parenting behaviors, such as maternal involvement in children’s education, is of vital importance. While confirming that working mothers are at a disadvantage in engaging in children’s education, the current study also attempted to identify the potential effects that work schedules have on parental involvement in children’s education. The study shows that the emergence of the 24/7 economy has encouraged a significant proportion of mothers in Hong Kong to engage in non-standard work (both hours and days) during the preschool years of children, a critical period for children’s long-term developmental path. However, the issue of non-standard work schedules has been largely ignored in public and academic discussions in Hong Kong. This is the first study to demonstrate that non-standard schedules are a significant predictor of maternal involvement in children’s education. It joins a small but growing body of literature which provides evidence that the timing of work is an important employment parameter for parenting and child wellbeing (Kaiser et al., 2017; Prickett, 2018; Haines et al., 2020; Hwang and Ramadoss, 2020; Zhao et al., 2021).

A unique contribution of the current study is that it differentiated the two dimensions of non-standard work schedules (i.e., working non-standard hours vs. weekends), and observed that they had disparate associations with maternal involvement. The study found that weekend work was negatively associated with home-based involvement. Specifically, mothers who *often* worked on weekends had lower levels of home-based involvement than weekday workers. This finding aligns with previous research which showed that weekend schedules are linked with higher ratings of work-family conflicts for parents (Laß and Wooden, 2021) and/or reduced work-family balance (Crawley, 2021). As evidenced by Craig and Brown (2014), parents, when working weekends, spend less time with children on those days. More importantly, they have limited abilities to recover this lost weekend time with children on weekdays (Craig and Brown, 2015; Laß and Wooden, 2021), because weekend work is often a result of overwork (Hook, 2012) (i.e., mothers working at weekends tend to have more working hours over the course of the week). This evidence on weekend work and time shared with children may also explain why similar negative associations were undetected for those *always* working weekends. In results not shown but available on request, it was found that mothers who always worked on weekends had significantly lower numbers of working hours (*M* = 37.0) than those who often worked weekends (*M* = 50.3). This difference in working hours suggests that mothers with regular weekend schedules are likely to have rest days on weekdays that can be used to compensate fully or partially for lost weekend time with children. For others, on the other hand, weekend work is likely to be a product of overwork, which may account for the lower levels of home-based involvement among mothers who often worked weekends.

In contrast to weekend work, working non-standard hours had a positive association with maternal involvement. Significantly, the level of school-based involvement by mothers working non-standard hours was greater than for those working regular daytime hours, and indeed comparable to the level for full-time mothers not in the labor force. This finding suggests that working non-standard hours can be used as a work option that allow mothers to use daytime hours for children (Kim, 2020b), which lends support to claims for the potentially positive aspect of non-standard work schedules (Taht and Mills, 2012). As Kim (2020b) pointed out, moreover, mothers’ satisfaction with non-standard hours enabling them to balance the competing responsibilities of employment and parenthood may override physical fatigue and mental stress from working non-standard hours, which may lead to positive outcome in terms of family functioning and children’s wellbeing. However, it should be noted that the findings also suggest that mothers are at a disadvantage in engaging in school involvement when they work regular daytime hours. In Hong Kong, indeed, school involvement events, such as volunteering activities and parent–teacher meetings, typically occur during the daytime and weekdays (Garvis et al., 2021). This scheduling practice of schools, which may explain why weekend work had no association with school-based involvement, may constrain working mothers’ ability to make school-involvement volunteer in the school or to attend parent–teacher meetings, if they work daytime hour.

It is worth noting that working non-standard hours did not entail negative influences on home-based involvement. This finding may come as a surprise, given the existence of a copious literature illuminating the undesirable parenting outcomes caused by working non-standard hours (Prickett, 2018; Haines et al., 2020), such as reductions in parental time with children (Lee et al., 2017; Tsai, 2022). A study by Enchautegui (2013), however, pointed out that these negative effects are dependent on the age of children. Her study found that reductions in parental time with children occurred only when mothers with school-aged children worked non-standard hours. By contrast, such reductions were not detected for mothers with preschoolers, which suggests that the effects of the daily timeframe of non-standard schedules (i.e., working non-standard hours) are not always negative. In this connection, it has also been shown that even if working non-standard hours reduces parental time with children, working mothers often attempt to compensate for lost time by reducing personal leisure and sleep (Wight et al., 2008; Prickett and Augustine, 2021). This compensation strategy, which often utilizes weekend time, can be translated into maternal efforts to maintain their involvement. For example, a recent study by Gracia and García-Román (2018) found that mothers working non-standard hours during weekdays increased the time they spent on children’s educational activities over the weekends. This explains not only why the level of home-based involvement by mothers working non-standard hours did not differ from that for standard schedule workers, but also why weekend work had a negative influence on home-based involvement, as shown above.

Finally, this study detected a different pattern of results with regard to the association between working hours and maternal involvement. As noted earlier, previous studies commonly reported a non-linear effect of maternal working hours, with part-time working mothers having greater levels of maternal involvement than full-time working mothers and those not in the labor force (Muller, 1995; Weiss et al., 2003; Youn et al., 2012). In the current study, however, those not in the labor force had the highest level of parental involvement. Though part-timers had higher levels of home-based involvement than mothers with long working hours, these associations disappeared after work schedule characteristics were controlled for. This pattern of results suggests that the effect of working hours might be spurious. In other words, the negative association between working hours and parental involvement may be simply because mothers working long hours worked during the weekend (Hook, 2012), while weekend work, paired with inadequate rest days during weekdays, might lead to adverse outcomes for home-based involvement. This finding adds to the literature demonstrating that *when* parents work matters more for parenting than the *total number* of hours they work (Gracia and García-Román, 2018; Zhao et al., 2021).

### Limitations

Although the study’s results are intriguing, some limitations should be noted. First, due to the cross-sectional nature of the data, it is difficult to disentangle the causal impact of maternal employment characteristics on parental involvement. The analytic models of the study controlled for an extensive set of maternal, family and child characteristics. In addition, sensitivity analyses, testing whether the inclusion of different sets of control variables alters findings, revealed that the findings of the study were robust. However, there may be unobservable factors that simultaneously affect respondents’ work characteristics and their involvement in children’s education (e.g., mothers’ intellectual ability). Causal interpretation should be made with caution. Second, the data analyses relied on the binary measure of non-standard work hours (standard vs. non-standard), due to small frequency issues. This procedure may mask the differential effects that the specific types of work schedules may have on parenting practice (Kim et al., 2016; Kim and Liu, 2021). Though it is occasionally argued that the broad variable more accurately captures the erratic or inconsistent characteristics of contemporary work schedules (Cho and Coulton, 2016), additional studies are still needed to examine how various types of non-standard hours influence parental involvement. Third, this study assessed work schedules in the *primary* job. It is possible that individuals may have a second job that often involves a non-standard schedule (Laß and Wooden, 2021), which may lead to underestimation of the prevalence of non-standard work schedules in Hong Kong. Last, while the current investigation assessed mothers’ work schedules and their involvement in children’s education, some scholars have asserted that fathers’ work schedules may have differential effects on parenting outcomes (Davis et al., 2006; Kim, 2021). Though research on fatherhood indicates that fathers have become similar to mothers in experiencing the dual demands of parenting and employment (Nomaguchi and Johnson, 2016), the gendered division of labor still prevails (García-Román, 2021), particularly in Hong Kong (Leung, 2018). Under this circumstance, fathers may not perceive the same pressures as mothers to recover missed time with children when working non-standard schedules, suggesting that negative parenting outcomes associated with non-standard work schedules might be more salient among fathers. However, the survey respondents who consisted predominantly of mothers made it impossible to examine SEM models that offer reliable estimates regarding the association between fathers’ work schedules and their involvement in children’s education. Future research is warranted to examine whether and how paternal work schedules affect paternal involvement, and thereby understand differences (or similarities) in the effect of maternal and paternal work schedules.

### Implications for Practice, Policy, and Future Research

Despite the limitations noted above, the results of the study offer pertinent implications for policy and practice. First, Hong Kong needs to consider legislation setting standard/maximum work hours and assuring adequate rest periods. In Hong Kong, currently, there is neither a specific policy nor a collective agreement regulating working hours/schedules (Zou and Leung, 2019), along the lines of the European Union’s Working Time Directive of 2003. Such a piece of legislation will ensure that parents who often work weekends have adequate rest periods during weekdays, which in turn allows them to be able to carve out time for their children. However, this would require high levels of consensus among multiple stakeholders, given its far-reaching socio-economic implications (Labour Department, 2012). At least in the short term, it is doubtful whether policies with such far-reaching implications for the labor market would be acceptable in an economy traditionally run along *laissez-faire* lines (Wong, 2012). As a results, it is necessary to simultaneously consider other policy options, such as flexitime, a work arrangement that allows employees to adjust their time of reporting and leaving work within certain limits (Chung and van der Horst, 2018). Such schedule controls have been identified as an important tool in protecting working parents, including weekend workers, from adverse parenting outcomes (Genadek and Hill, 2017; Laß and Wooden, 2021). Efforts by companies in Hong Kong to offer flexible work arrangements for their employees, however, have been relatively few, despite the spur to remote working given by the COVID-19 pandemic (Choi et al., 2020; Wong et al., 2021). More direct forms of government intervention (e.g., subsidies or tax incentives) may be needed to motivate employers to adopt family-friendly scheduling practices.

Another strategy is to intervene directly with working parents. For instance, practitioners may develop and provide parenting programs targeting parents working weekends and/or working regular daytime hours to mitigate the adverse consequences of their work schedules. A promising option is Workplace Triple P, a variant of Triple P (Positive Parenting Program) specifically aimed at working parents (Sanders et al., 2011). Guided by cognitive-behavioral and social learning principles, Workplace Triple P has been shown to be effective in reducing work-family conflicts and enhancing parenting efficacy among working parents (Hartung and Hahlweg, 2011; Haslam et al., 2013). Though it has yet to be used in the local context, the demonstrated effectiveness of the original Triple P for Hong Kong parents (Leung et al., 2003, 2014) suggests that Workplace Triple P is also likely to benefit working parents in Hong Kong (Kim, 2021), where practitioners are ready to offer the evidence-based parenting program (Kim, 2019a, 2020a).

A final set of strategies pertains to schools that can play an important role in addressing challenges from non-standard work schedules. While the importance of parental involvement has been recognized in Hong Kong (Ho and Kwong, 2013; Wu and Sun, 2020), school administrators often request parental involvement without taking into consideration the workplace needs of working parents. In an effort to accommodate working parents’ needs, for example, schools may consider offering daytime and evening slots when holding parent–teacher conferences. Online parent–teacher conferences, which have been frequently organized during the COVID-19 pandemic, should also be helpful. In addition, schools may consider providing greater social/emotional support and intellectual stimulation aimed at children with parents who work weekends and thereby may have a limited capacity for parental involvement (Li et al., 2014).

In addition to policy/practice implications, the current study illuminates avenues for future research as well. Notably, the study may shed light on future efforts to examine the mechanism through which parental work schedule affect family members. Recognizing that parental experiences at the workplace may spill over to the family, a growing body of research has examined the association between parental work schedules and child wellbeing (Kaiser et al., 2017; Kachi et al., 2021). In the Chinese context, for instance, recent studies by Han (2020) and Kim (2021) showed that parental non-standard work schedules were associated with various developmental outcomes for young children in Hong Kong and mainland China, respectively. Previous research, however, has tended to focus on the direct linkage. Our understanding about the pathways through which parental work schedules affect children still remains limited. Given the findings of the study, future research is needed to investigate whether parental involvement in children’s education may serve as a mediator that links parental work schedules and child outcomes in the Chinese contexts.

## Conclusion

As Hong Kong moves toward a 24/7 economy, working non-standard schedules is becoming an increasingly pervasive feature of its workforce. This trend is unlikely to be reversed in the near future, given the projected job growth of service-sector occupations in which non-standard schedules are relatively common. This study is the first study to demonstrate the importance of considering the temporal pattern of maternal working hours in understanding the interplay between employment and parenting outcomes in the Chinese context. While non-standard schedules involves both the hourly and weekly time frames, the findings suggest that working non-standard schedules is linked to both positive and negative qualities of maternal involvement in children’s education, depending on whether mothers work non-standard hours or work weekend. These findings, indicating that the effects of non-standard work schedules are not uniformly negative or positive, warrant sophisticated policy efforts to address the potential adverse effects of non-standard work schedules and avoid undermining their potential to be used as a family-friendly option.

## Data Availability Statement

The original contributions presented in this study are included in the article/supplementary material, further inquiries can be directed to the corresponding author.

## Ethics Statement

The studies involving human participants were reviewed and approved by the Survey and Behavioural Research Ethics Committee of the Chinese University of Hong Kong. The patients/participants provided their written informed consent to participate in this study.

## Author Contributions

MK and NJ designed the research, conducted the data analyses, and wrote the first draft of the manuscript. LW conducted the literature reviews and descriptive data analyses. All authors read and approved the final manuscript.

## Conflict of Interest

The authors declare that the research was conducted in the absence of any commercial or financial relationships that could be construed as a potential conflict of interest.

## Publisher’s Note

All claims expressed in this article are solely those of the authors and do not necessarily represent those of their affiliated organizations, or those of the publisher, the editors and the reviewers. Any product that may be evaluated in this article, or claim that may be made by its manufacturer, is not guaranteed or endorsed by the publisher.
